# Recapping and mite removal behaviour in Cuba: home to the world’s largest population of Varroa-resistant European honeybees

**DOI:** 10.1038/s41598-022-19871-5

**Published:** 2022-09-16

**Authors:** Anais Rodríguez Luis, Isobel Grindrod, Georgiana Webb, Adolfo Pérez Piñeiro, Stephen John Martin

**Affiliations:** 1 Centro de Investigaciones Apícolas, La Habana, Cuba; 2 School of Science, Engineering and Environment, The University of Salford, Manchester, M5 4WT UK

**Keywords:** Ecology, Evolution

## Abstract

The *Varroa destructor* ectoparasitic mite has spread globally and in conjunction with Deformed Wing Virus has killed millions of honeybee (*Apis mellifera*) colonies. This has forced Northern hemisphere beekeepers into using miticides to avoid mass colony losses. However, in many Southern hemisphere countries widespread treatment did not occur since miticides were prohibitively expensive, or a centralised choice was made not to treat, both allowing natural selection to act. The Varroa mite initially caused high losses before mite-resistance appeared in the honeybee populations. Initially, mite-resistance was only associated with African and Africanised honeybees. Although recently, several isolated mite-resistant European honeybee populations have appeared. Here we studied the mite-resistance in Cuba and found high rates of recapping of infested worker cells (77%), high removal of mites (80%) and corresponding low mite fertility (*r* = 0.77). These are all traits found in all naturally evolved Varroa-resistant populations. We can confirm Cuba has the world’s largest European mite-resistant population with 220,000 colonies that have been treatment-free for over two decades and illustrating the power of natural selection. Cuban honeybees are also highly productive, 40–70 kg of honey produced annually, and are mild mannered. Cuba is an excellent example of what is possible when honeybees are allowed to adapt naturally to Varroa with minimal human interference.

## Introduction

During the past 70 years the Varroa mite (*Varroa destructor)*, in association with Deformed Wing Virus (DWV) that it transmits, has spread world-wide, killing millions of *Apis mellifera* colonies, particularly in the Northern Hemisphere. This resulted in the almost universal uptake of miticides in the Northern Hemisphere to control Varroa populations. However, in some Southern Hemisphere countries e.g., Mexico, Brazil, and Africa the beekeepers either could not afford to treat^1^, do not treat as they consider Varroa not a threat^2^, or their central beekeeping organisation decided not to treat when Varroa arrived^3^ but allowed natural selection to run its course. Initially, these countries suffered high losses e.g., South Africa^3^, but these losses declined after several years as honeybees adapted to the mite and became Varroa-resistant. No initial losses were recorded in Brazil when the resistant Africanised bees spread, but any initial losses may have been masked by the losses of Varroa-infested European honeybee colonies. On a much smaller scale a growing number of European^4,5^ and Hawaii^6^ beekeepers are taking a similar approach of ceasing treatment or collecting resistant feral colonies. A recent study^7^ found that all well-established resistant populations, despite being widely dispersed across several continents, all share the same key traits, which all arise from the ability of the workers in resistant colonies to detect mite-infested cells using chemical signals produced by the mite offspring^8^. This leads to increased rates of recapping and removal of infested cells, which leads to the death of all mite offspring in those cells, and it is this that reduces the mites’ ability to reproduce and promotes increased mite infertility. Subsequently, there is a long-term decrease of mite populations and viral loads, whilst there is an increase in colony survival rates^7^. The role of elevated recapping rates has been consistently found in the majority of self-selected honeybee populations^4,6,9^, with one known exception^10^. Although both recapping and mite removal can be highly variable traits^7^ they are currently the best ‘proxy’ for a resistant population since recapping is spatially associated with the presence of infested cells^11^.

The largest Caribbean Island is Cuba, being 1250 km long, covering 109,884 km^2^ and currently containing over 220,000 managed colonies. In the 1950’s Cuban colonies were estimated at between 100,000 and 150,000 when Eva Crane visited Cuba in 1957^12^. In 1968 managed colonies were censused and this revealed 151,000 hives. During the 1970’s and 1980’s beekeeping continued to grow with State support to 208,000 colonies in 1985. After this colony numbers fluctuated, reaching a minimum of 126,000 colonies in 2003. This was due to Varroa and an economic crisis. Since then, colony numbers have steady increased, reaching 221,000 colonies in 2021. All the managed colonies are currently kept by 1,900 government registered beekeepers that have always selected for productivity, hygienic behaviour and calmness, under the Centro de Investigaciones Apícolas (CIAPI) Bees Selection Program. As a result, Cuban bees are highly productive: annually averaging 45–70 kg of honey per colony according to honey production records held by CIAPI and have 80% hygienic behaviour, based on removal of dead brood. In addition, a large unmanaged feral honeybee population exists due to expansive regions of flowers and the Cuban Royal palm (*Roystonea regia*) forests that cover around 25% of Cuba.

Honeybees (*A. m. mellifera*) were first introduced into Cuba from the USA (Florida) in 1768, followed later by *A. m. ligustica*, *A. m. caucasica* and *A. m. carnica*^13^. Despite the presence of Africanised honeybees in some, but not all, surrounding Caribbean Islands^14^, a honeybee import ban for the last 60 years has allowed this large European population to thrive in Cuba. Studies using allozyme markers^15,16^ confirmed that the Cuban honeybee population is European. Later this was re-confirmed by using mitochondrial haplotypes (microsats) which mostly belonged to European lineages (e.g., M, and C)^17^. In addition, the microsatellite data showed that Cuba has a homogeneous population of managed honeybees across the country without any regional differences, confirming the isolated nature of the population.

Despite the 60-year honeybee import ban, in 1996 Varroa was first detected in Matanzas province in Western Cuba and further investigations found the mite in and around La Habana city. The mite was predicted to have entered Cuba a couple of year’s earlier^18^, potentially via shipping or illegal queen imports. In 1997 around 8000 colonies died, all infested with Varroa, and by 1998 the mite had spread to the seven western provinces. Movement of bees between major regions was prohibited, some mite control via drone-brood trapping was used, but more losses were suffered before Varroa resistant bees appeared, several years after the mite invaded Cuba. Thereafter, no mite treatments have been administered for over 20 years^19^ making the Cuban population the largest Varroa-resistant European honeybee population in the world.

Previous research^20^ found DWV is in 100% of apiaries and only the Korean haplotype of Varroa was found in Cuba^19^. Therefore, the situation in Cuba, with respect to the bees, mites, and virus, is similar to that found across the Northern hemisphere. Therefore, the aim of this study was to investigate if the traits found in other resistant populations from other countries i.e., increased recapping and mite removal, along with reduced ability of Varroa to reproduce, were present in the worker brood of the Cuban honeybee population. In addition, recapping and mite reproduction was measured in drone brood.

## Results

### Honeybee data

In December 2021 a total of 6,923 worker and 1,906 drone brood cells were investigated from the 32 and 16 colonies respectively that were managed in six locations across western Cuba (see “Methods and materials”—Fig. 5). The average Varroa-infestation rate of worker (13% SD ± 10%) and drone (40% ± SD 18%) capped brood was significantly different (d.f. = [15, 31], *t* = 6.8909 *p* = 0.00001) as expected.

### Recapping data

**Figure 1:**
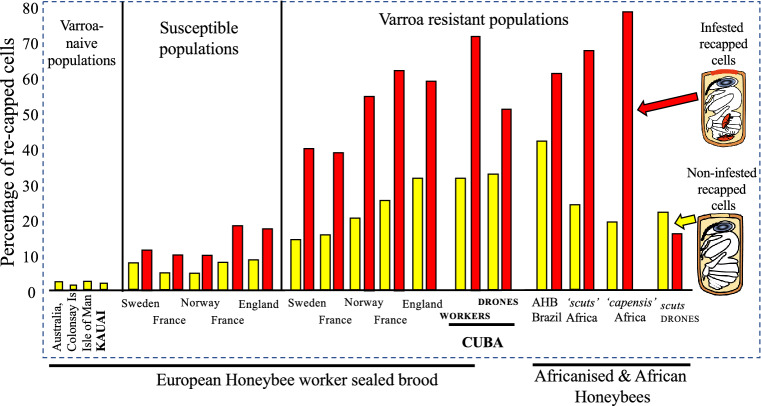
Recapping rates from both worker and drone showing levels in non-infested (yellow) or infested (red) cells from this (in bold) and previous studies^4,5,9^.

To provide baseline data, 3,090 worker brood cells from five Varroa-naïve colonies from Kauai, Hawaii had a mean recapping rate of 3.6% (± 4.2 SD) and median of 1.3% due a single outlier colony in which the recapping rate was 10.7%. In comparison, the average recapping rate in Cuba for infested worker brood was 72% (± 21SD), while non-infested cells were recapped at 33% (± 33SD), which again is significantly different (df_30,24_ *t* = − 6.9304, *p* = 0.00001). Whereas, in the 1,906 drone brood the recapping levels of 52% (infested) and 34% (non-infested) were not significantly different (df _10,10_, *t* = − 1.7208, *p* = 0.101) due to the high level of inter-colony variability (Fig. 1).

**Figure 2:**
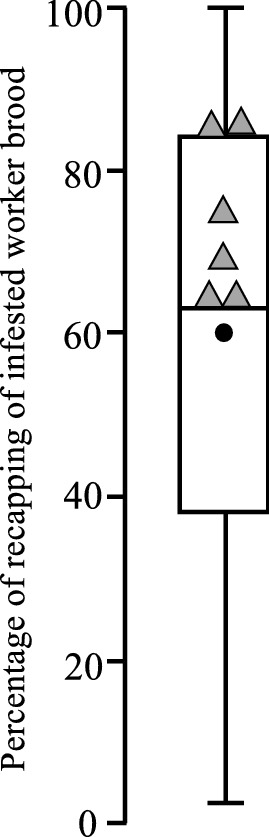
A comparison of the percentage of recapping of infested cells from the six Cuban locations (triangles) in comparison with the 101 data points from the other resistant *A. mellifera* colonies studied around the world according to^7^ is shown as a box plot.

The weighted average recapping rate of infested worker brood for all previous studies is 55% in mite resistant colonies and 33% in susceptible colonies^7^. Whereas the weighted average for all the Cuban colonies is 63%. Hence, each of the six locations in Cuba can be classed as highly resistant, since all six locations were above the average and median of all previous studies (Fig. 2).

**Figure 3:**
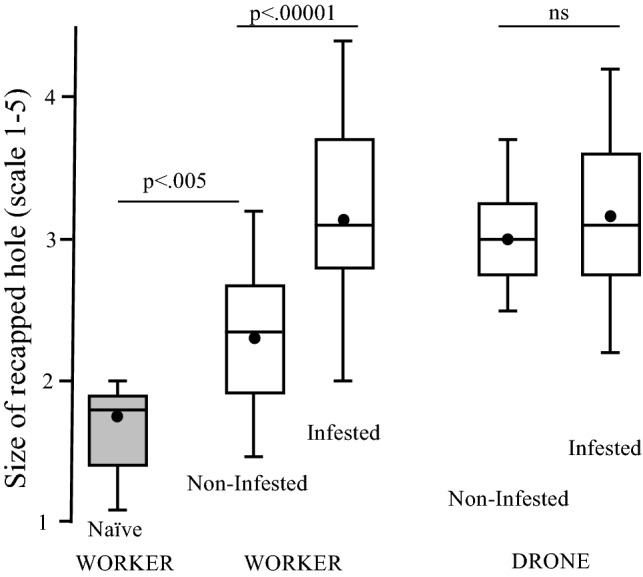
Size of recapping holes in Varroa-naïve worker cells (Kauai) compared with Cuban workers and drones capped brood that were infested or not.

The diameter of the recapped hole (p < 0.005) is significantly smaller in the baseline Varroa-naïve Kauai colonies than Cuban worker cells non-infested colonies (mean = 1.7 mm, SD ± 0.34, vs. mean = 2.3 mm, SD ± 0.45). Whereas recapped sizes in infested cells (mean = 3.2 mm SD ± 0.69) were significantly larger than non-infested worker brood, although there was no significant difference in recapping size between infested and non-infested drone brood (mean = 3.2 mm, SD ± 0.68 vs. 3.0 mm, SD ± 0.41) (Fig. 3).

The average colony recapped size of Varroa-naïve worker cells, Kauai, were significantly smaller than non-infested Cuban worker capped brood (d.f [4, 30], *t* = 2.981, *p* = 0.0054). Whereas there was a significant larger recapped area in infested cells, than non-infested cells (df_24, 29_ *t* = 5.53239, *p* = 0.00001) in Cuban worker brood. However, in drone capped brood, no significant difference in recapped size (df_10,10_ *t* = − 0.8728, *p* = 0.196) was found between non-infested and infested cells.

### Mite removal

**Figure 4:**
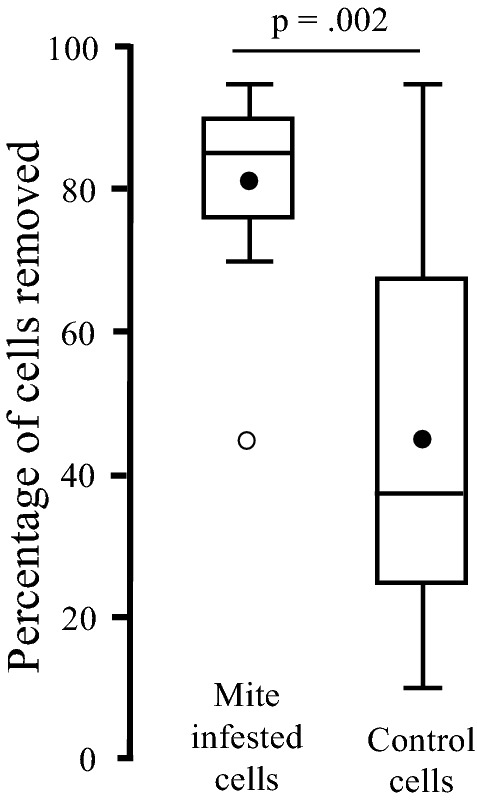
Percentage of removal in mite infested and control cells indicating the significant difference between the two groups and showing a large inter-colony variation among the control cells, relative to the mite infested cells.

In March 2022 a total of 200 artificial mite introductions and 200 control sham openings were performed on worker brood from ten colonies in the CIAPI apiary. There were significantly more artificially mite infested cells removed than sham control openings (df _9,9_ *t* = -3.5135, *p* = 0.00248). In fact, over 35% more (Fig. 4), since 81% of the mite-infested cells were removed while 45% of the controls were removed. Of the 38 mite-infested not removed 36 (95%) were recapped. While of the 111 control cells not removed 80 (72%) were recapped. The removal rates of the mites were consistent (except one outlier) across the ten colonies. The removal of control cells was highly variable (Fig. 4). In 14 (7%) cells the mite was missing, although since they all had been recapped, they probably escaped during the period the cell was open. A total of 26 mites (23%) were found in the remaining 111 control cells at the end of the experiment.

### Varroa mite data

Data on mite-reproduction was collected from 688 worker and 350 drone single infested capped brood cells that aged from white-eye (85 h post-capping) to the resting stage. Of these, 195 workers and 57 drones were grey pad (240 h post-capping) or older. In addition, 31 workers and 46 drones were infested by two or more mites and were grey pad (300 h post-capping in drones) to resting stage and were used for reproductive success calculations. Overall, an average of 0.77 new viable (mated) offspring were produced in worker cells and 1.6 in drone cells (Table 1).

**Table 1:** Various Varroa reproductive classifications in Cuban worker and drone capped brood cells.

Classification	Worker	Drones
Viable mothers > po	72%	71%
Non-repo mothers > po	8%	11%
Viable offspring > gp live male + moulted skin	51%	65%
Viable offspring > gp in multple-infested cells	32%	49%
Total fertility in all cells > gp	47%	53%
Reproductive rate > gp single infested cells	0.87	1.96
Reproductive rate > gp multiple infested cells	0.49	1.42
Total Reproductive rate in all cells > gp	0.77	1.6

The minimum pupal age category is also given. Pale eyes (po) ≈ 100 h in workers and 120 h in drone. Grey pads (gp) ≈ 240 h in workers and 300 h in drones. The moulted skin of male mites is used to confirm they are adults.

The number of viable offspring produced in singly infested worker cells aged 240 h post-capping, or older in recapped (mean = 0.88) or non-recapped (mean = 0.85) cells, were not significantly different (*t*_133, 60_ = 0.18276, *p* = 0.855). The result is the same when all infested worker cells (single and multiple) are considered (*t*_157, 67_ = 0.71157, *p* = 0.477), indicating recapping per se has no direct effect on mite mortality.

## Discussion

We confirm that Cuba is home to the world’s largest European honeybee population that has naturally become Varroa-resistant, with an estimated 220,000 colonies being maintained without any form of chemical treatment for over two decades^19^ although some drone-trapping occurred during the early years of the transition period This is despite the presence of the K-haplotype of the mite^20^ and the widespread occurrence of DWV^19^ throughout Cuba. Hence, the Cuban honeybee population is the first major case of Varroa-resistant European bees occupying an entire country of a large size (109,884 km^2^). In Europe the proportion of varroa-resistant honeybee populations in each country is highly variable^21,22^, but they still consist of small, isolated populations within any country. For example, the second largest known area of European Varroa-resistant honeybees is in North Wales, UK where 104 beekeepers have managed around 500 honey bee colonies over an area of 2500 km^2^ without treatment for over a decade^23^.

It has long been established that sub-Sharan African and Africanised honeybees are Varroa-resistant and both populations cover much larger areas than Cuba, but these honeybee races are not capable of thriving in temperate regions or are rejected by beekeepers in Northern hemispheres. However, previous studies on African/Africanised and European honeybees^4–6,9^ all appear to have evolved with the same resistance mechanism^7^ and Cuban honeybees follow this pattern showing high recapping behaviour, high mite removal behaviour and low mite reproduction (Figs. 1, 4, Table 1).

The strongest evidence that increased recapping behaviour is a direct response to the presence of Varroa, is the very low recapping rates in Varroa-naïve colonies. This is evidenced by the recapping baseline data that has now been collected from four different Varroa-naïve (Varroa free) honeybee populations (Australia, UK [two populations] and Hawaii [this study]) all producing similar results (Fig. 1). Across the four populations, a total of 9542 worker cells from 15 colonies have been studied with an average recapping rate of 2.0% (+ SD 3.2). Interestingly, only two of the colonies had atypical recapping rates of 8.5% and 10.7%, from Australia and Kauai respectively. This may suggest increased sensitivity in these colonies as no obvious causes e.g., wax moth or dead pupa, were detected in either colony. The data summary in Fig. 1 indicates that even in Varroa-treated populations the workers are still able to detect mite infested cells, but the average consistently falls significantly below that found in resistant populations. That is, in non-infested worker cells recapping rates are significantly higher in resistant populations in comparison to susceptible populations (Fig. 1) *t*_4, 5_ = − 4.185, *p* = 0.0023 as well as for infested cells *t*_4, 5_ = − 6.905, *p* = 0.00007.

The ability of Cuban honeybees to detect infested cells causes not only high recapping levels but also high removal rates of artificially mite-infested cells. A mean removal rate of 81% is among one of the highest recorded in *Apis mellifera*^7^. The average control rate of 45% is driven by three colonies that all removed more than 75% of the controls, while the average of the remaining seven colonies was 28%. During the mite-removal studies in March 2022 natural Varroa infestation was 23%, whereas in December 2021 it was only 13%. This is due to decreasing worker brood rearing, caused by a shortage of nectar during the annual dry season. During this time there is an increase in hygienic behaviour in the colonies^24^, which could help explain the higher-than-expected removal of control cells.

The reproductive ability of Varroa to produce viable i.e., mated, female offspring (*r*) in infested worker cells in resistant colonies in South Africa^4^ (*r* = 0.9), Brazil^4^ (*r* = 0.8), Mexico^18^ (*r* = 0.73), Europe^3^ (*r* = 0.84) is similar to the 0.87 found in Cuba (this study). In Cuba ‘*r*’ reduces to 0.77 when both single and multiple infested cells are considered. This reduction in mite reproduction, relative to susceptible colonies that have values of *r* greater than one, is directly linked to the increased ability of resistant workers to both detect and remove, by cannibalisation, the infested pupa. Hence, this ensures the invading mite fails to reproduce^7^ or reduces mite fertility due to the recapping process^4^. Although, in this study no significant difference was found in the reproduction of Varroa in recapped or non-recapped cells, supporting the findings of two previous studies^5,9^. Therefore, recapping may be playing a minor role in resistance. However, recapping remains the best indicator or ‘proxy’ of resistance within the vast majority of honeybee populations since it’s easier, quicker, and it requires less skill to measure recapping rates than mite removal rates. However, recapping is a highly variable trait^7^, hence both many cells (200–300) per colony and many colonies (> 10) per population ideally need to be studied to help reduce the variablity, also in temperate countries measuring recapping when mite-infestation rates peak in autumn maximises detecting infested cells since the recapping of cells is spatially associated with infested cells^11^.

Despite the current focus on what is happening in worker cells, studies focusing on the role of recapping in drone brood are still in their infancy with. Currently, data is only available from South Africa^9^ (Fig. 1) and now Cuba (this study). Interestingly, both studies indicate no significant difference in recapping rates between infested and non-infested brood. This is caused by some colonies performing no recapping of drone brood, while some colonies do recap cells but in a non-targeted manner. Whereas there is a significant increase in the size of the recapped area between infested (3.1 mm) and non-infested (2.3 mm) worker cells (Fig. 3), this does not occur in drone brood, as it appears that the holes are entirely exploratory. However, the lack of removal of infested drone brood may be playing an important role in mite-resistance (see below).

The mite infestation of worker cells currently varies between 23 and 13% in Cuba (this study), roughly 25 years after it was first detected (1996). Whereas, in Mexico and Brazil, infestation rates of worker brood have fallen from around 20% in 1996/1999 down to 4% in 2018/19^7^. Although, Varroa was first detected in Brazil much earlier, in 1972^25^ and the Africanised honeybees adapted to the mite and spread northward replacing the susceptible European colonies. Therefore, we predict that the worker infestation rate in Cuba will continue to fall over the next 20 years, especially if high mite-removal rates persist. Correspondingly, we would expect to see the infestation rates of the drone brood (currently at 40%) to remain high as mites potentially avoid reproduction in worker cells. This potentially is a key, but currently overlooked part, of the resistance mechanism. Since an empirical model^26^ indicated that negative mite population growth occurs in (resistant) Africanised honeybee colonies only when the initial drone cells are present. This is thought to arise because mites also show a tenfold preference to reproduce in drone cells (which comprises only 1–5% of all the honeybee brood) and they soon become overcrowded as the mite population increases. This leads to inter-mite competition for the limited food and space, causing an increase in mite mortality^27^, resulting in negative reproductive success for mites entering these overcrowded drone cells. Thus, mite population growth in drone brood cells is limited by a density-dependent mechanism. In Cuba it has been observed that strong colonies typically with drone brood do not weaken during the drought season, whereas colonies without drone brood are weak and often die during the drought (APP personal comm).

Although Cuban beekeepers have been aware of their mite-resistant honeybees for 15 to 20 years’, Cuba’s situation has only recently come to light^16,18^. The main reason for Varroa-resistance in Cuba is due to the centralised decision to allow natural resistance to evolve, as also was done successfully in South Africa^3^, rather than becoming locked into using miticides, as has happened throughout the Northern hemisphere. The CIAPI and Veterinarian Services central decision to ‘not treat’ was greatly assisted by all Cuban beekeepers being professional, registered and embedded within a strong locally based beekeeping community where colony movement and exchange of queens is within each province.

There is also a large feral population and due to Cuba’s sub-tropical climate, queens are replaced annually in managed colonies because of almost continuous egg-laying, similar to honeybees in Hawaii. This rapid queen turnover speeds up natural selection relative to honeybee populations in more temperate climates. Finally, Cuba’s 60-year ban on honeybee importation has helped isolate the country from been invaded by Africanised bees which has occurred in many nearby regions (eg. Mexico, Southern USA, Puerto Rico, neighbouring Dominican Republic^13^ and Haiti (D. Macdonald, Apiary Inspector, Min. of Agi BC, Canada, pers. Comm.). Cuba has many managed European colonies coupled with many queen rearing stations. These colonies are productive and mild mannered. Thus, Cuba is an excellent example of the power of natural selection in honeybees when they are allowed to adapt naturally to Varroa with minimal human interference.

## Methods and materials

### Recapping study

**Figure 5:**
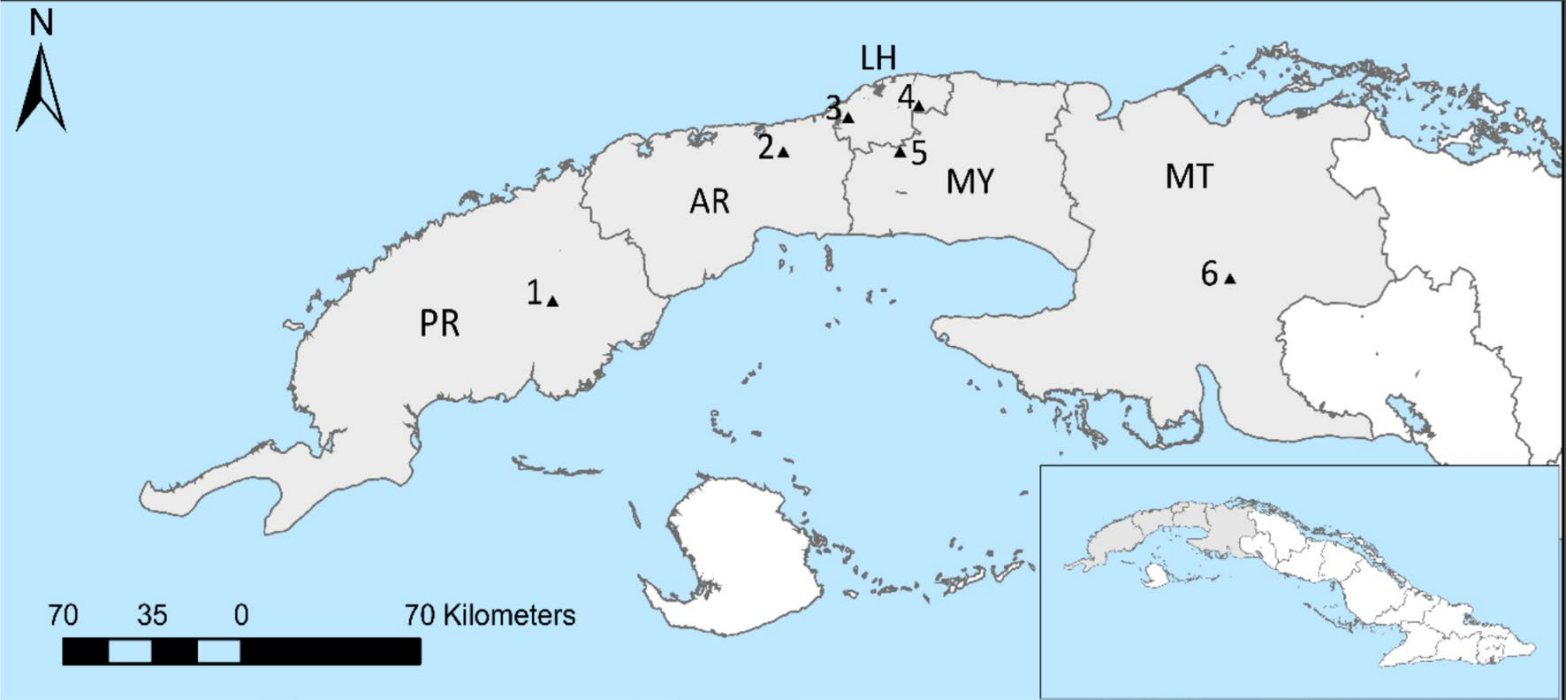
Black triangles represent the approximate locations of the six sampling apiaries in Western Cuba. Letters indicate the name of the provinces: Pinar del Rio (PR), Artemisa (AR), La Habana (LH), Mayabeque (MY), Matanzas (MT).

A total of 37 colonies were sampled in December 2021 from six locations spread across 250 km of Western Cuba (Fig. 5). From each colony a patch of capped worker or drone brood, if present, containing approximately 300 cells was cut out of the frame. In the laboratory using binocular microscopes and ring lights, we investigated if each cell had been recapped, by carefully inverting the cell cap and estimating the size of the recapping on a scale from 1 to 5, which in worker brood equates roughly to a 1–5 mm scale. The age of the pupae was recorded using changes in eye or body colour^28^ and if infested each of the mite stages were recorded^28^. Mite exuviate indicated the presence of an adult male or female and was important in determining multiple invaded cells and the number of mated female offspring. No baseline data can be collected in Cuba since no Varroa-free regions exist. Therefore, baseline recapping rates were collected from the Varroa-free colonies from the Island of Kauai (Hawaii) since it lies at a similar latitude to Cuba, has a similar sub-tropical climate and is home to European honeybees. From each of five Kauai colonies around 600 worker brood cells per colony were studied for recapping rate and size of recapped area estimated on the 1–5 scale.

### Mite removal study

The removal rates of ten colonies from CIAPI (Fig. 5, location 3) were studied during March 2022. For each colony 20 mites were collected from capped drone brood within 2–3 days of capping and the mother mites were individually placed using a fine paint brush into 20 worker capped brood cells that were within 1–2 days of been capped over. In addition, 20 control sham openings were also conducted. The positions of all the manipulated cells were recorded on an acetate sheet. The logic behind using mites from young drone brood is they will have already received the stimulus to start egg laying^29^ since the mite-offspring produce the compounds detected by the worker honeybees^8^. The artificially infested frames were immediately reintroduced into the origin colonies. Eight days after the mites were inserted, the ten frames were removed and the number of infested and control cells that were removed were recorded, along with the recapping levels of unremoved cells.

### Data analysis

For each colony we measured the recapping rate and hole size of infested and non-infested cells in all cells older than 85 h post-capping (white eye stage), the brood infestation rate using all pupae. These values were then standardised by calculating percentages. However, if fewer than 5 mite-infested cells were present in a colony then the data on infested recapped cells and the size of recapped cells were excluded, to avoid the effects of a small sample size. All data was then averaged across all colonies and values compared to previous studies. As the resulting recapping and mite-removal data were all normally distributed (Kolmogorov–Smirnov Test of Normality), parametric statistics are used throughout.

To ensure a sufficient sample size, we pooled the mite data from all the colonies knowing that all Cuban honeybees are considered genetically similar^16^. Both single and multiple infested worker or drone cells reproductive values were calculated separately to allow direct comparisons with previous studies. The mite development figures^30,31^ were used to check if the development timings of Varroa found in Cuba are similar to that found previously, which they were.

## Data Availability

All raw data will be available on request from the corresponding author.
